# Disparities in end-of-life care, expenditures, and place of death by health insurance among cancer patients in China: a population-based, retrospective study

**DOI:** 10.1186/s12889-020-09463-1

**Published:** 2020-09-04

**Authors:** Zhong Li, Peiyin Hung, Ruibo He, Xiaoming Tu, Xiaoming Li, Chengzhong Xu, Fangfang Lu, Pei Zhang, Liang Zhang

**Affiliations:** 1 Department of Social Medicine and Health Management, School of Medicine and Health Management, Tongji Medical College, Huazhong University of Science and Technology, Wuhan, Hubei China; 2 Department of Health Services Policy and Management, Arnold School of Public Health, University of South Carolina, Columbia, SC USA; 3 Department of Labor and Social Security, School of Finance and Public Administration, Hubei University of Economics, Wuhan, Hubei China; 4 Department of Intelligent Computing and Mathematics, School of Biomedical Engineering and Informatics, Nanjing Medical University, Nanjing, Jiangsu China; 5 Department of Health Promotion, Education, and Behavior, Arnold School of Public Health, University of South Carolina, Columbia, SC USA; 6 Yichang Center for Disease Control and Prevention, Yichang, Hubei China; 7 Research Center for Rural Health Service, Key Research Institute of Humanities & Social Sciences of Hubei Provincial Department of Education, No. 13 Hangkong Road, Wuhan, Hubei China

**Keywords:** End-of-life care, Expenditures, Place of death, Health insurance, Cancer patients

## Abstract

**Background:**

Disparities in the utilization, expenditures, and quality of care by insurance types have been well documented. Such comparisons have yet to be investigated in end-of-life (EOL) settings in China, where public insurance covers over 95% of the Chinese population. This study examined the associations between health insurance and EOL care in the last six months of life: outpatient visits, emergency department (ED) visits, inpatient services, intensive care unit (ICU) admissions, expenditures, and place of death among the cancer patients.

**Methods:**

A total of 398 patients diagnosed with cancer who survived more than 6 months after diagnosis and died from July 2015 to June 2017 in urban Yichang, China, were included. Descriptive analysis and multivariate regression models were used to investigate the bivariate and independent associations, respectively, between health insurance with EOL healthcare utilization, expenditures and place of death.

**Results:**

Urban Employee Basic Medical Insurance (UEBMI) beneficiaries visited EDs more frequently than Urban Resident-based Basic Medical Insurance (URBMI) and New Rural Cooperative Medical Scheme (NRCMS) beneficiaries (marginal effects [95% Confidence Interval]: 2.15 [1.81–2.48] and 1.92 [1.59–2.26], respectively). NRCMS and UEBMI beneficiaries had more hospitalizations than URBMI beneficiaries (1.01 [0.38–1.64] and 0.71 [0.20–1.22], respectively). Compared to URBMI beneficiaries, NRCMS beneficiaries and UEBMI beneficiaries had ¥15,722 and ¥43,241 higher expenditures. Similarly, UEBMI beneficiaries were most likely to die in hospitals, followed by NRCMS (UEBMI vs. NRCMS: 0.23 [0.11–0.36]) and URBMI (UEBMI vs. URBMI: 0.67 [0.57–0.78]) beneficiaries.

**Conclusions:**

The disproportionately lower utilization of EOL care among NRCMS and URBMI beneficiaries, compared to UEBMI beneficiaries, raised concerns regarding quality of EOL care and financial burdens of NRCMS and URBMI beneficiaries. Purposive hospice care intervention might be warranted to address EOL care for these beneficiaries in China.

## Background

As the leading cause of death in China, new cancer cases totalled 4.3 million in 2015, and 2.8 million cancer patients died that year [1], a rapid increase compared with an estimated 3.8 million new cancer cases and 2.3 million cancer patient deaths in 2014 [2]. Cancer has posed great challenges to the Chinese healthcare system, such as accessibility and affordability of treatment and hospice care [3], patient engagement [4] and quality of death [5]. However, primary care facilities have limited access to opioid drugs, insufficient training and a shortage of specialists and generally do not provide hospice care [6]. Moreover, hospice care provided by the primary care facilities or nursing facilities is still hampered by reimbursement policies [6]. Unbearable end of life (EOL) symptoms force patients or their families to seek help from hospitals, and an increasing number of patients have to die in hospitals [5]. One prior study revealed that, in addition to China’s 400 cancer hospitals, only a few charity hospitals and primary care facilities provided hospice care [7]. An official report showed that less than 1% of hospitals provided hospice care services in 2016 in China [8].

China ranked in the last quartile of countries worldwide on the quality of death, according to the 2015 Quality of Death Index proposed by the Economist Intelligence Unit [7]. With the increasing number of cancer survivors, the disparities in access to EOL care and the intensity of EOL healthcare utilization have been widely debated [9, 10]. Several randomized trials among end-stage cancer patients showed that earlier palliative care could lead to improved quality of life and less aggressive care [11, 12]. Despite the nationwide strategic procurement for cancer care in China, historical studies revealed that expenditures per admission among cancer patients varied by cancer type and health insurance [13, 14]. In other words, higher socioeconomic status (SES) led to higher expenditures [3]. The Global Atlas of Palliative Care at the End of Life also revealed that income levels are generally positively associated with the availability and quality of palliative care [15]. In high-income countries, low SES is a risk factor for in-hospital death and increased utilization of acute EOL healthcare services [16]. One prior study also showed that Medicaid beneficiaries or self-insured populations were observed to have fewer admissions to hospice care within 3 days of death due to unbearable out-of-pocket expenditures [17]. Adolescents and young adults with cancer living in low-income areas were less likely to receive hospice enrolment [18]. One prior study of cancer patients showed that 62.4% of them died in hospital settings in China from 2015 to 2017, higher than the 46.1% of cancer patients in England estimated to have died in hospital settings during the years 2001–2010 [19]. Studies in China have shown that risk factors for in-hospital deaths include high SES [14], absence of hospice care or nursing facilities, and shorter survival after diagnosis [5]. Two studies of cancer patients showed that free medical treatment occupied a large proportion of health care resources in China [20, 21]. However, the two studies were conducted in the 1990s and may not demonstrate current practice patterns [20, 21].

Typically, EOL care refers to all the care provided within the last few months of a patient’s life. Palliative care focuses on improving the quality of life and care for patients with serious illness by preventing and relieving suffering, managing pain and other physical, psychosocial and spiritual problems, and communicating with the patients and their families about expectations of care. However, the illness is not yet considered to be a terminal illness. For hospice care, patients (and/or their families) know that they are not expected to recover from their illness. Generally, treatment for the illness, such as life-prolonging medications, will not be pursued [22, 23]. With the Basic Standards for Hospice Care (for Trial Implementation) and Practice Guidelines for Hospice Care (for Trial Implementation) issued by the Chinese government [5], pioneering hospice care programmes are scaling up, including inpatient hospice care, continuous hospice care, hospice at home, respite care delivered by hospitals, hospice care centres, and primary care facilities. Hospice interdisciplinary care teams, including physicians, nurses, social workers, volunteers, hospice aides and bereavement specialists, jointly provide management of patients’ illnesses and symptoms, as well as emotional and spiritual care for patients and their families [5]. However, only a few local governments issued targeted reimbursement policies for hospice care. Lack of EOL care guidelines might also lead to various healthcare utilizations and intensive EOL care and expenditures, exacerbating the current inequalities in the quality of EOL care [24]. In fact, aggressive EOL care has caused skyrocketing medical expenditures during the last months of patients’ lives, aggravating the existing financial burden of the health system [25].

In China, outside of the Urban Employee Basic Medical Insurance (UEBMI) scheme, nearly 100% of rural and urban residents have been enrolled in the New Rural Cooperative Medical Scheme (NRCMS) and Urban Resident-based Basic Medical Insurance (URBMI) schemes, respectively [26]. The UEBMI scheme has more comprehensive service coverage and financial protection than the other two schemes [26]. Outpatient services were generally not covered by the NRCMS and URBMI schemes [26]. Many rural residents moving to urban areas are still enrolled in the NRCMS scheme [26]. Fragmented health insurance schemes have caused inequitable access to healthcare [26]. Previous studies showed disparities in reimbursement or expenditures for tuberculosis healthcare services [27], for cancer patients [13] and for the elderly [28]. Moreover, as the current payment system is oriented towards a curative approach, physicians tend to provide more aggressive care, which leads to more curative treatment and later hospice enrolment [8]. National reimbursement policies specific for hospice care have also not been enacted [6]. However, disparities in utilization, expenditures and quality of care by insurance types persist. The estimation of disparities in utilization of healthcare services caused by different health insurance types is urgently needed so that the hospice care system can be strengthened.

Currently, studies on the Chinese hospice care system have mainly focused on advance care planning, nursing, traditional healthcare utilization, expenditures and place of death (POD) [29], overlooking the relationship between health insurance and EOL care utilization, expenditures and POD. Moreover, the Chinese hospice care system is still in its infancy [7, 8, 29]. Different provinces have their own processes regarding health insurance consolidation. How reimbursement policies should be implemented to incentivize hospitals and physicians to provide preferable EOL care for patients is unknown, even though this information is critical for policymakers and healthcare administrators. Therefore, this study aimed to compare EOL healthcare utilization, expenditures, and POD among the three universal health insurance schemes in China.

## Methods

### Data collection

In Yichang, China, the policy on health insurance consolidation was issued in June 2017 and implemented in January 2018 [30]. By 2018, over 23.3% of the population was over 60 years of age. However, current hospice care is provided mainly in inpatient settings; no specific hospice care facilities are available. Hospice care services are generally reimbursed by traditional fee-for-service or global budget due to the lack of reimbursement packages for hospice care. Based on the International Statistical Classification of Diseases, 10th Revision (ICD-10), we first identified decedents from July 2015 to June 2017 from the National Population Death Registration and Management System. We then extracted cancer patients from the Yichang Health Management Data Center. After integrating the two databases, 894 cancer patients were initially identified in Yichang, China [3, 5, 31]. A total of 398 cancer patients who survived more than 6 months after diagnosis were ultimately included in the current study.

### Variables

The independent variable was the type of health insurance. Each decedent’s health insurance type was identified from electronic health records collected by the Yichang Health Management Data Center. Outcomes of interest consisted of EOL healthcare utilization during the last 6 months of life, including number of emergency department (ED) visits, number of outpatient visits, number of hospital admissions and whether a decedent had at least one intensive care unit (ICU) admission, as well as total healthcare expenditures during the last 6 months of life, and POD (in-hospital or home death) [3, 5, 31]. Any spending by patients during the ED visits, outpatient visits, inpatient services, and ICU admissions were included from the supply side. In the current study, as the database included only data from the hospitals in Yichang, we did not include EOL care and expenditures among the primary care facilities in Yichang or hospitals out of Yichang. We included the following confounders in our analysis: gender (male and female), age (< 65, 65–80, > 80), marital status (previously married or single, currently married), education attainment (primary school or less, senior school, college or above), and survival time (6–12 months, > 12 months) [3, 5, 31].

### Statistical analysis

EOL expenditures were first adjusted to present values in 2017 using the consumer price index based on the Yichang Health Statistics Yearbook. Chi-squared or Fisher exact tests were used to compare differences in SES characteristics by insurance type for categorical variables. The Kruskal–Wallis test followed by Dunn’s pairwise comparison was used for count variables and continuous variables. As expenditure data were skewed distributed, generalized linear models with a gamma distribution and log link function were employed to estimate the associations between health insurance and EOL care expenditures. For the number of outpatient visits, ED visits, and inpatient admissions, generalized linear models with a Poisson distribution and log link function were conducted. For the outcome of POD, a generalized linear model with a binominal distribution and logit link function was conducted. The marginal effect facilitates the estimation of diverse outcomes for defined groups with the change in the independent variables, and we reported the average marginal effect to interpret the data [32]. Moreover, to characterize the potential unmeasured confounding needed to explain the current results, we calculated E-values for each adjusted odds ratio and relative risk [33, 34]. In this study, the E-value estimates what the relative risk would have to be for any unmeasured confounder to overcome the observed association of health insurance type with EOL care, expenditures and POD. All procedures were conducted with Stata 14.0. *P* <  0.05 was considered to indicate statistical significance.

## Results

### Basic characteristics by health insurance

As shown in Table 1, among the 398 patients, 64.8% were male, 65.6% were elderly (≥ 65), and 85.9% were married. Nine percent of them attained at least one college degree. A total of 45.0% of the patients survived longer than 12 months after diagnosis. URBMI (65.0%) beneficiaries and UEBMI (71.3%) beneficiaries consisted of more male patients than NRCMS (42.9%) beneficiaries (***χ***^***2***^ = 21.0, *P* <  0.001). URBMI beneficiaries were younger than the UEBMI group (***χ***^***2***^ = 13.7, *P* <  0.001). Regarding the level of education attainment, URBMI beneficiaries obtained a lower level of education than those from the NRCMS and UEBMI groups (***χ***^***2***^ = 23.4, *P* <  0.001). UEBMI (51.3%) beneficiaries had a higher ratio of survival for more than 12 months than URBMI (28.3%) and NRCMS (36.4%) beneficiaries (***χ***^***2***^ = 13.2, *P* = 0.001). Nearly one-third (33.2%) of patients were diagnosed with lung cancer.

**Table 1:** Distribution of patients’ characteristics by health insurance

Variable	Group	Overall	URBMI (1)	NRCMS (2)	UEBMI (3)	***χ***^***2***^	***P***	Post Hoc
		Number (Column %)						
**Gender**	Male	258 (64.8)	39 (65.0)	33 (42.9)	186 (71.3)	21.0	< 0.001	1 > 2, 3 > 2
	Female	140 (35.2)	21 (35.0)	44 (57.1)	75 (28.7)			
	< 65	137 (34.4)	32 (53.3)	30 (39.0)	75 (28.7)	14.4	0.006	1 < 3
**Age**	65–80	202 (50.8)	20 (33.3)	37 (48.1)	145 (55.6)			
	> 80	59 (14.8)	8 (13.3)	10 (13.0)	41 (15.7)			
**Marital**	Previously married or single	56 (14.1)	10 (16.7)	16 (20.8)	30 (11.5)	4.6	0.099	\
**status** ^**a**^	Currently married	342 (85.9)	50 (83.3)	61 (79.2)	231 (88.5)			
**Education**	≤ Primary school	285 (71.6)	57 (95.0)	59 (76.6)	169 (64.8)	23.4	< 0.001	1 < 2, 1 < 3
**attainment** ^**b**^	Senior school	76 (19.1)	3 (5.0)	12 (15.6)	61 (23.4)			
	≥ College	37 (9.3)	0 (0)	6 (7.8)	31 (11.9)			
**Survival** ^**c**^		334 (239, 454)	274 (209, 386)	285 (234, 405)	362 (253, 492)	15.5	< 0.001	1 < 3, 2 < 3
	6–12 months	219 (55.0)	43 (71.7)	49 (63.6)	127 (48.7)	13.2	0.001	1 < 3, 2 < 3
	> 12 months	179 (45.0)	17 (28.3)	28 (36.4)	134 (51.3)			
**Cancer type**	Lung (C34.x)	132 (33.2)	19 (31.7)	18 (23.4)	95 (36.4)	21.0	0.179	
	Stomach (C16.x)	31 (7.8)	4 (6.7)	4 (5.2)	23 (8.8)			
	Colorectum (C18.x, C19.x, C20.x)	41 (10.3)	7 (11.7)	6 (7.8)	28 (10.7)			
	Liver (C22.x)	46 (11.6)	5 (8.3)	10 (13)	31 (11.9)			
	Pancreas (C25.xl)	15 (3.8)	3 (5)	1 (1.3)	11 (4.2)			
	Biliary tract (C23.x, C24.x)	8 (2.0)	1 (1.7)	1 (1.3)	6 (2.3)			
	Prostate (C61.x)	9 (2.3)	2 (3.3)	1 (1.3)	6 (2.3)			
	Breast (C50.x)	18 (4.5)	2 (3.3)	8 (10.4)	8 (3.1)			
	Others (C00.x-C15.x,	98 (24.6)	17 (28.3)	28 (36.4)	53 (20.3)			
	C17.x, C21.x,							
	C26.x, C30.x-C33.x,							
	C37.x-C41.x, C43.x-C49.x,							
	C51.x-C58.x,							
	C60.x, C62.x,							
	C80.x, C88.x,							
	C90.x, C96.x, C97.x)							

*NRCMS* New Rural Cooperative Medical Scheme; *UEBMI* Urban Employee Basic Medical Insurance; *URBMI* Urban Resident-based Basic Medical Insurance; a, b: Fisher’s exact tests for cells < 5; c: Median (p25, p75) was reported, the Kruskal–Wallis test followed by Dunn’s pairwise comparison was used

### EOL healthcare utilization, expenditure and POD by health insurance

As shown in Table 2, UEBMI beneficiaries utilized more outpatient services than URBMI beneficiaries (U = 1.72, *P* = 0.043) and NRCMS beneficiaries (U = 4.02, *P* <  0.001). UEBMI beneficiaries had more ED visits than URBMI beneficiaries (U = 5.79, *P* <  0.001) and NRCMS beneficiaries (U = 6.36, *P* < 0.001). UEBMI beneficiaries also utilized more inpatient services than URBMI beneficiaries (U = 2.38, *P* = 0.009). Differences in ICU admission between beneficiaries from the three health insurance schemes were not statistically significant (*P* = 0.560). For the expenditures during the last 6 months, UEBMI beneficiaries spent more than URBMI beneficiaries (U = 6.11, *P* < 0.001) and NRCMS beneficiaries (U = 4.12, *P* < 0.001). NRCMS beneficiaries also spent more than URBMI beneficiaries (U = 1.98, *P* = 0.024). UEBMI beneficiaries (81.6%) experienced the greatest rate of in-hospital deaths, followed by NRCMS beneficiaries (54.5%) and URBMI (13.3%) beneficiaries (U = 107.1, *P* < 0.001).

**Table 2:** EOL care, expenditures and place of death by the health insurance

Variable	Overall	URBMI (1)	NRCMS (2)	UEBMI (3)	U/***χ***^***2***^	***P***	Post Hoc
	Median (p25, p75)						
**OS** ^**a**^	5 (1, 13)	5 (1.5, 10)	2 (0, 8)	6 (2, 14)	17.0	0.002	1 < 3, 2 < 3
**ED** ^**b**^	1 (0, 3)	0 (0, 1)	0 (0, 1)	2 (1, 4)	61.4	< 0.001	1 < 3,2 < 3
**IS** ^**c**^	3 (1, 5)	2 (1, 4)	3 (1, 5)	3 (2, 5)	5.6	0.062	1 < 3
**ICU** ^**d**^							
**Yes**	25 (6.3)	2 (3.3)	4 (5.2)	25 (7.3)		0.560	\
**No**	373 (93.7)	58 (96.7)	73 (94.8)	242 (92.7)			
**EOL expenditures** ^**e**^	31,349 (10,721, 77,801)	11,123 (610, 24,492)	19,953 (6871, 44,352)	45,389 (18,094, 93,156)	45.8	< 0.001	1 < 2 < 3
**POD** ^**f**^					107.1	< 0.001	
**Hospital**	263 (66.1)	8 (13.3)	42 (54.5)	213 (81.6)			1 < 2 < 3
**Home**	135 (33.9)	52 (86.7)	35 (45.5)	48 (18.4)			

*NRCMS* New Rural Cooperative Medical Scheme; *UEBMI* Urban Employee Basic Medical Insurance; *URBMI* Urban Resident-based Basic Medical Insurance; *EOL* End of life; *OS* Outpatient services; *ED* Emergency department; *IS* Inpatient services; *ICU* Intensive care unit; *POD* Place of death;

a: The Kruskal–Wallis test followed by Dunn’s pairwise comparison was used. 1 < 3: U = 1.72, *P* = 0.04, 2 < 3: U = 4.02, *P* < 0.001

b: The Kruskal–Wallis test followed by Dunn’s pairwise comparison was used. 1 < 3: U = 5.79, *P* < 0.001, 2 < 3: U = 6.36, *P* < 0.001

c: The Kruskal–Wallis test followed by Dunn’s pairwise comparison was used. 1 < 3: U = 2.38, *P* = 0.009

d: Number (Column %) was reported, Pearson’s Chi-squared test was used

e: The Kruskal–Wallis test followed by Dunn’s pairwise comparison was used. 1 < 2: U = 1.98, *P* = 0.024, 1 < 3: U = 6.11, *P* < 0.001, 2 < 3: U = 4.12, *P* < 0.001

f: Number (Column %) was reported, Pearson’s Chi-squared test was used. 1 < 2: ***χ***^***2***^ = 24.7, *P* < 0.001, 1 < 3: ***χ***^***2***^ = 106.0, *P* < 0.001, 2 < 3: ***χ***^***2***^ = 23.5, *P* < 0.001

### Marginal effect estimation

Table 3 presents the results of the marginal analysis comparing different outcomes among the three health insurance schemes. Based on the average marginal effect, UEBMI beneficiaries used 1.90 and 2.71 more outpatient services than URBMI (*P* < 0.001, 95% CI: 1.04 to 2.76) and NRCMS (*P* < 0.001, 95% CI: 1.98 to 3.43) beneficiaries, respectively. UEBMI beneficiaries were observed to utilize 2.15 more ED visits (*P* < 0.001, 95% CI: 1.81 to 2.48) and 1.92 more ED visits (*P* < 0.001, 95% CI: 1.59 to 2.26) than URBMI and NRCMS beneficiaries, respectively. For the inpatient services, NRCMS beneficiaries and UEBMI beneficiaries utilized 1.01 (*P* = 0.002, 95% CI 0.38 to 1.64) and 0.71 more inpatient services (*P* = 0.006, 95% CI: 0.20 to 1.22) than URBMI beneficiaries, respectively. Regarding EOL expenditures, NRCMS beneficiaries and UEBMI beneficiaries spent 15,721.8 more Chinese Yuan (*P* = 0.005, 95% CI: 4685.3 to 26,758.3) and 43,241.2 more Chinese Yuan (*P* < 0.001, 95% CI: 31914.1 to 54,568.4) than URBMI beneficiaries, respectively. Additionally, UEBMI beneficiaries spent 27,519.5 more Chinese Yuan (*P* < 0.001, 95% CI: 13978.6 to 41,060.3) than NRCMS beneficiaries. In addition, the likelihood of in-hospital death was 44% (*P* < 0.001, 95% CI: 29 to 58%) greater for NRCMS beneficiaries than for URBMI beneficiaries. The likelihood of in-hospital death was 67% (*P* < 0.001, 95% CI: 57 to 78%) greater for URBMI beneficiaries than for UEBMI beneficiaries. The likelihood of in-hospital death was 23% (*P* < 0.001, 95% CI: 11 to 36%) greater for UEBMI beneficiaries than NRCMS beneficiaries. Results of these regression models, controlling for gender, age group, marital status, education attainment, and survival, are presented in Table 4.

**Table 3:** Marginal effect estimates for the health insurance schemes

Variable	OS	ED	IS	EOL expenditures	POD
	Estimates (95% CI)				
NRCMS (vs. URBMI)	−0.80 (−1.77, 0.16)	0.22 (− 0.13,0.58)	1.01 (0.38, 1.64) **	15,721.8 (4685.3, 26,758.3) **	0.44 (0.29, 0.58) ***
UEBMI (vs. URBMI)	1.90 (1.04, 2.76) ***	2.15 (1.81–2.48) ***	0.71 (0.20, 1.22) **	43,241.2 (31,914.1, 54,568.4) ***	0.67 (0.57, 0.78) ***
UEBMI (vs. NRCMS)	2.71 (1.98, 3.43) ***	1.92 (1.59, 2.26) ***	−0.30 (− 0.82, 0.22)	27,519.5 (13,978.6, 41,060.3) ***	0.23 (0.11, 0.36) ***

*NRCMS* New Rural Cooperative Medical Scheme; *UEBMI* Urban Employee Basic Medical Insurance; *URBMI* Urban Resident-based Basic Medical Insurance; *EOL* End of life; *OS* Outpatient services; *ED* Emergency department; *IS* Inpatient services; *POD* Place of death. *, *P* < 0.05: **P < 0.01; *** *P* < 0.001

**Table 4:** Associations between health insurance and EOL care, expenditures and place of death

Variable	OS ^a^	ED ^b^	IS ^c^	EOL expenditures ^d^	POD ^e^
	IRR (95% CI)	IRR (95% CI)	IRR (95% CI)	Exp (beta) (95%)	AOR (95%)
NRCMS (vs. URBMI)	0.89 (0.78, 1.02)	1.26 (0.87, 1.83)	1.37 (1.12, 1.67) **	1.80 (1.21, 2.70) **	9.12 (3.65, 23.02) **
UEBMI (vs. URBMI)	1.26 (1.12, 1.40) ***	3.50 (2.58, 4.73) ***	1.26 (1.05, 1.50) ***	3.21 (2.26, 4.56) ***	30.09 (12.56, 72.11) **
UEBMI (vs. NRCMS)	1.41 (1.27, 1.56) ***	2.77 (2.17, 3.55) ***	0.92 (0.80, 1.06)	1.77 (1.30, 2.43) ***	3.28 (1.82, 5.92) ***

*NRCMS* New Rural Cooperative Medical Scheme; *UEBMI* Urban Employee Basic Medical Insurance; *URBMI* Urban Resident-based Basic Medical Insurance; *EOL* End of life; *OS* Outpatient services; *ED* Emergency department; *IS* Inpatient services; *POD* Place of death; *IRR* Incidence rate ratio; *AOR* Adjusted odds ratio. Controlling for gender, age group, marital status, education attainment, and survival. * *P* < 0.05 ** P < 0.01 *** *P* < 0.001. a: generalized linear models with a Poisson distribution and log link function; b: generalized linear models with a Poisson distribution and log link function; c: generalized linear models with a Poisson distribution and log link function; d: generalized linear models with a gamma distribution and log link function; e: generalized linear models with a binominal distribution and logit link function. The marital status variable was dichotomized into 1) currently married and 2) previously married or never married (single)

### Sensitivity analysis

The E-value estimates what the relative risk would have to be for any unmeasured confounder to overcome the observed association of health insurance type with EOL care, expenditures and POD in this study, enabling us to interpret how strong the confounding would have to be to explain away our estimate and, thus, how much evidence we truly have for association. The E-values for the point estimates and lower 95% confidence bounds for EOL care, expenditures and POD by health insurance type are presented in Table 5. In this study, an E-value of 17.73 would indicate that an unmeasured confounder would have to increase both the likelihood of shifting from URBMI to NRCMS and the likelihood of in-hospital death by 17.73-fold each if the type of health insurance was to have no association with in-hospital death.

**Table 5:** E-values for health insurance schemes and EOL care, expenditures and place of death

Variable	OS ^**a**^	ED ^**b**^	IS ^**c**^	EOL expenditures ^**d**^	POD ^**e**^
	E-value (lower 95% confidence bound)				
NRCMS (vs. URBMI)	1.50 (1.00)	1.83 (1.00)	2.08 (1.48)	3.00 (1.71)	17.73 (6.76)
UEBMI (vs. URBMI)	1.83 (1.49)	6.46 (4.60)	1.83 (1.28)	5.87 (3.95)	59.68 (24.61)
UEBMI (vs. NRCMS)	2.17 (1.86)	4.98 (3.76)	1.39 (1.00)	2.94 (1.92)	6.02 (3.04)

*NRCMS* New Rural Cooperative Medical Scheme; *UEBMI* Urban Employee Basic Medical Insurance; *URBMI* Urban Resident-based Basic Medical Insurance; *EOL* End of life; *OS* Outpatient services; *ED* Emergency department; *IS* Inpatient services; *POD* Place of death

## Discussion

To the best of our knowledge, as the first study to explore the disparities in EOL care, expenditures and POD among the three health insurance schemes, this study enriches the current literature on the research of Chinese health insurance reform, specifically in hospice care reimbursement policies. Huge disparities in EOL care utilization, expenditure and POD exist, revealing the great potential to improve the hospice care system in China. The findings of this study also highlight the urgent need to enact targeted reimbursement policies for EOL care, thus reducing EOL aggressive care and healthcare expenditures.

First, UEBMI beneficiaries visited outpatients and EDs more frequently than URBMI beneficiaries and NRCMS beneficiaries. In addition, NRCMS beneficiaries and UEBMI beneficiaries were observed to have a greater utilization of inpatient services than URBMI beneficiaries. These results may be related to the fact that UEBMI had a higher reimbursement ratio. URBMI and NRCMS beneficiaries had to utilize inpatient services for outpatient services that were not covered by the URBMI benefit package, but with a limited reimbursement ratio [35]. In addition, NRCMS beneficiaries had a higher level of education attainment than URBMI beneficiaries, which generally means a higher level of income and affordability for EOL care [36]. However, the current data do not allow us to include income in our analysis. Given the fact that residents from rural areas generally had a lower level of education attainment than their urban counterparts [37]. This result might also be related to the fact of small sample size of this study. The current results did not find differences in ICU admissions among the three schemes. Given the fact that a higher level of reimbursement ratio was not associated with ICU admissions, we may cautiously infer that public awareness of avoiding unnecessary aggressive care might have been raised. However, one previous study also revealed that public awareness of advance care planning is not enough [38]. A comparative study between Ontario, Canada, and the United States revealed that limited availability of hospice care in Ontario and different attitudes towards EOL care might be related to different EOL care utilization [39]. Compared to beneficiaries with Medicare and Medicaid Hospice Benefits, beneficiaries with private insurance, particularly those in managed care plans, demonstrated disparities in terms of the hospice benefits they received [40]. Hence, we can cautiously infer that higher proportions of inpatient services might be used due to the inadequate supply of home- or community-based hospice care and the lack of specific reimbursement policies. Lessons from Taiwan’s National Health Insurance system also showed that determining the insurance coverage and reimbursement level for specific services and providing more incentives for institutions will help promote home- or hospital-based hospice care [41].

Second, UEBMI beneficiaries spent more than beneficiaries in the other two schemes. Our results indicated that a higher proportion of out-of-pocket expenditures or stricter policies might cause disparities in EOL care utilization. It might be related to the fact that UEBMI and URBMI beneficiaries used more hospital-based services [42]. A retrospective cohort study revealed that expanded hospice care benefits to end-stage renal disease patients are associated with a 7.3% reduction in EOL expenditures [43]. Therefore, hospice care consultation should be delivered to patients in advance, which is associated with reduced EOL aggressive care utilization and expenditures [44, 45]. The future reimbursement policy should also encourage patients or their families to engage in more advance care planning with the providers under updated preferences and goals of EOL care [44–46]. Notably, well-educated patients could accurately understand do-not-resuscitate orders [16]. Such a policy might be applicable to UEBMI beneficiaries who are generally of a higher SES.

Third, more than 80% of UEBMI beneficiaries died in hospitals. This result contradicts results of one prior study in Ontario, Canada, revealing that patients who lived in higher-income urban communities had a greater likelihood of dying at home [46]. This finding reflects the large gap between the patients’ preference and the actual POD situation in urban China. Therefore, it is vital for patients and policy makers to raise awareness through discussion of the preferred POD [36]. Moreover, the greater proportion of UEBMI beneficiaries who died in hospitals revealed a high intensity of EOL care utilization. This outcome is consistent with the findings of one prior study that revealed that higher SES led to a higher possibility of in-hospital death [47]. These results revealed that a large proportion of healthcare resources might be wasted, hindering the system-building of community-based or home-based hospice care. Given the fact that less than 1/3 of Chinese oncologists preferred to refer cancer patients to hospice care [6], these huge disparities also highlight the great potential to optimize the current hospice care system. As a high-quality death was associated with the professional support received by the patients’ caregivers, EOL discussions and conversations are urgently needed to help improve the possibility of earlier hospice care services and reduced expenditures. Finally, advance planning should be introduced and enhanced to reduce engagements with patients or their families because the current system requires the physician to coordinate with patients’ families when an emergency occurs, which would mean unnecessary expenditures and in-hospital deaths.

Our study has several limitations. First, while the current study was representative of cancer patients in urban Yichang, it may not be generalized to the whole population in China. Second, we could not obtain direct expenditures from the primary care facilities or institutions outside of Yichang or patients’ or their families’ income variables, so they are not considered in the current study. Given that primary care facilities generally do not provide any hospice care services, home-based hospice care might not be available for many patients, increasing the likelihood of patients’ dying in hospitals. Therefore, we should compare our results with findings based on other countries more cautiously. Third, the current database did not collect stages of cancer or whether the EOL care included hospice care and its duration; we should consider the time of hospice care enrolment and the patients’ actual utilization of hospice care in our analyses. Fourth, due to the limitations of the sample size, we could not conduct a stratified analysis or use a matching method to reduce the effect of selection bias. We were also unable to control for the variations in EOL care by hospital-level characteristics and practice patterns.

## Conclusion

In conclusion, EOL healthcare utilization, expenditures and POD for cancer patients vary within different health insurance schemes. Lack of purposive reimbursement policies and clinical guidelines might have caused patients or their families to choose inpatient services as a substitute for professional hospice care services. The results of the current study could help future studies investigate more urgent evidence to help explain influential factors, thus guiding the system-building of the hospice care system from the perspective of health insurance. To achieve the goal of quality EOL care, an urgent goal is to establish a specific reimbursement policy and EOL consultation for cancer patients in China. Intensive hospice care from outpatient services—both from primary care facilities and hospitals—and inpatient services by primary care facilities may be a promising approach to ensure the availability of more appropriate hospice care services that are relatively affordable.

## Data Availability

Raw data of the current study was provided by the Yichang Health Management Center affiliated with the Yichang Center for Disease Control and Prevention per the cooperation agreement between the Yichang Center for Disease Control and Prevention and School of Medicine and Health Management, Huazhong University of Science and Technology. As the raw data were de-identified by the research team, de-identified data used during the current study are available from the corresponding author upon reasonable request.

## Abbreviations

EOL: End of life
POD: Place of death
SES: Socioeconomic status
UEBMI: Urban employee basic medical insurance
NRCMS: New rural cooperative medical scheme
URBMI: Urban resident-based basic medical insurance
ICD-10: International statistical classification of diseases, 10th revision
ED: Emergency department
ICU: Intensive care unit
CI: Confidence interval
